# Distinct growth of the nasomaxillary complex in *Au. sediba*

**DOI:** 10.1038/srep15175

**Published:** 2015-10-15

**Authors:** Rodrigo S. Lacruz, Timothy G. Bromage, Paul O’Higgins, Viviana Toro-Ibacache, Johanna Warshaw, Lee R. Berger

**Affiliations:** 1 Dept. Basic Science and Craniofacial Biology, New York University College of Dentistry, New York, NY 10010, USA; 2 Depts. of Biomaterials & Biomimetics, New York University College of Dentistry, New York, NY 10010 USA; 3 Centre for Anatomical and Human Sciences, Hull York Medical School, University of York, York, YO10 5DD, UK; 4 Facultad de Ondontología and Facultad de Medicina, Universidad de Chile; 5 Evolutionary Studies Institute, University of the Witwatersrand, Private Bag 3,Wits 2050, South Africa

## Abstract

Studies of facial ontogeny in immature hominins have contributed significantly to understanding the evolution of human growth and development. The recently discovered hominin species *Autralopithecus sediba* is represented by a well-preserved and nearly complete facial skeleton of a juvenile (MH1) which shows a derived facial anatomy. We examined MH1 using high radiation synchrotron to interpret features of the oronasal complex pertinent to facial growth. We also analyzed bone surface microanatomy to identify and map fields of bone deposition and bone resorption, which affect the development of the facial skeleton. The oronasal anatomy (premaxilla-palate-vomer architecture) is similar to other *Australopithecus* species. However surface growth remodeling of the midface (nasomaxillary complex) differs markedly from *Australopithecus*, *Paranthropus*, early *Homo* and from KNM-WT 15000 (*H. erectus/ergaster*) showing a distinct distribution of vertically disposed alternating depository and resorptive fields in relation to anterior dental roots and the subnasal region. The ontogeny of the MH1 midface superficially resembles some *H. sapiens* in the distribution of remodeling fields. The facial growth of MH1 appears unique among early hominins representing an evolutionary modification in facial ontogeny at 1.9 my, or to changes in masticatory system loading associated with diet.

Immature fossil hominins are rare, especially those with completely preserved skulls and faces. The recovery of the nearly complete face of a juvenile of *Australopithecus sediba* (MH1) and the remarkable preservation of this specimen offers the prospect of extending our knowledge of hominin facial ontogeny and the evolution of human facial form. MH1 shows a unique set of derived facial characteristics relative to other *Australopithecus*^1^,^2^,^3^. Here we examine the facial morphogenesis of MH1 focusing on key aspects of its anatomy impacting on its growth including the oronasal complex and the remodeling of the midfacial skeleton^4^,^5^. We show that MH1 presents distinctive features of facial growth, retaining *Australopithecus*-like anatomical relations of the vomer and premaxilla but presents unique facial remodeling activity relative to any other fossil hominin taxon sampled to date. We consider how this developmental modification in the facial ontogeny of MH1 might relate to morphological differences with other hominins, to diet and its possible effects on loading.

The oronasal complex, in particular the anatomical relations of the intranasal bones and premaxilla, have been shown to vary among *Australopithecus*, *Paranthropus* and *Homo*^6^,^7^,^8^. This variation has been posited as impacting on facial morphogenesis principally on palatal thickening through a physical morphogenetic interaction between the anterior extension of the vomer and the premaxilla^5^. To assess these anatomical relations we examined MH1 using high energy (synchrotron) CT imaging and compared its anatomy to the conditions found among other hominins.

Facial remodeling is also an important process affecting the ontogeny of the face^9^,^10^,^11^,^12^,^13^,^14^,^15^,^16^,^17^,^18^,^19^. Remodeling involves the coordinated deposition of bone matrix by osteoblasts and the resorption of bone by osteoclasts resulting in the balanced growth of the skull. This occurs in concert with changes in the size, shape and relative positions (displacement) of individual cranial skeletal elements during postnatal growth and concomitant development and eruption of the dentition^7^,^8^,^9^,^10^,^11^,^12^,^13^,^14^,^15^,^16^,^17^. This balanced growth of the skull is determined by the integration of the individual skeletal units into functional components^8^,^14^,^15^,^16^,^17^,^18^. Thus, growth remodeling sculpts and adapts the developing facial skeleton. Different facial forms therefore show temporal and spatial differences in the rates and modes of remodeling activity^12^,^15^,^18^,^19^,^20^,^21^. Mapping and comparing such activity among distinct species provides insights into the cellular mechanisms that underlie differences in facial growth and thus into how changes in development contributed to evolutionary modifications in cranial form among hominins^12^,^15^,^18^,^19^,^20^,^21^. For MH1, high-resolution replicas of the face were examined in the scanning electron microscope (SEM) (see Methods) and surface remodeling features were mapped. These were compared with previously described remodeling features of the skulls of diverse hominins and other primates, and with the findings of a finite elements analysis of incisor biting in a modern human to assess potential mechanical signals for remodeling activity.

## Results

### Oronasal anatomy

The juvenile MH1 facial skeleton is nearly complete but matrix fills its intranasal spaces preventing direct observation of the relations of maxilla, premaxilla, nasal floor and vomer. High-energy beam synchrotron imaging allowed us to non-invasively investigate this aspect of the internal anatomy of MH1 and compare it with other hominins. MH1 shows a step-like relationship between the premaxilla and the palate (similar to the continuous-discrete classification of ref. 6) and the vomer does not contact the premaxilla (Fig. 2A, Figs S3, S4). It shares these features with *Au. afarensis* and *Au. africanus.* This common arrangement of the oro-nasal complex leads to the expectation that MH1 might also share a common spatial and temporal growth remodeling map. This was investigated by mapping and comparing remodeling activity over the face of MH1 with maps from other hominins.

### Facial growth remodeling map

**Figure 1:**
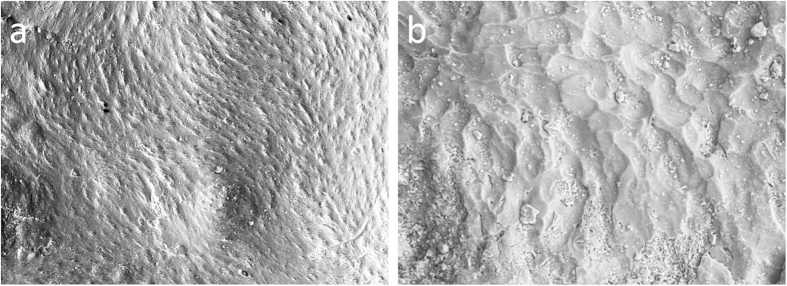
Electron micrographs of bone microanatomical features. Scanning electron micrographs of bone deposition **(a)** and resorption **(b)** from high-resolution replicas made of the MH1 face.

**Figure 2:**
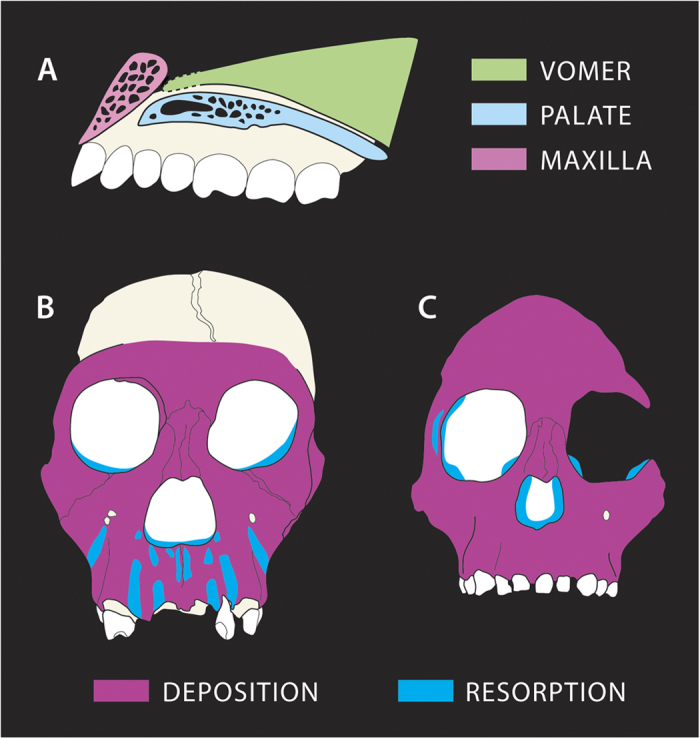
Facial characteristics of MH1. (**a)** Diagrammatic representation of the individual components of the intranasal region of MH1 based on synchrotron data (See also Figs S3 and S4). A step-like (similar to continuous-discrete classification of ref. 4) relationship between premaxilla and nasal cavity floor can be identified in MH1 as well as a lack of contact of the premaxilla with the vomer. (**b)** Reconstructed facial growth remodeling map of the face of MH1. Bone deposition is indicated by magenta whereas bone resorption is indicated in blue. Resorption can be observed along various portions of the lower face most predominantly along the alveolar region. (**c)** Reconstructed facial map of *Australopithecus* (*Au. afarensis* + *Au. africanus*) superimposed on Taung’s face (reproduced from ref. 12) based on the analysis of the sub-adult specimens LH 2, AL 333-105, LH 21, Sts 2, Stw 59, Taung, Sts 24, Sts 57, MLD 2 and Sts 52. Drawing of skull in b) by the authors from original photographs. Skull on c) drawn by the authors with permission from TGB.

The excellent preservation of the bone surfaces of the MH1 facial skeleton permitted a detailed reconstruction of depository and resorptive fields (Fig. 1, Figs S1, S2). Both the left and right side of the maxillae independently provided a nearly continuous record of bone microstructural detail. Such pristine surface details are often found in specimens fossilized within the South African cave breccias or tufa environments. Contrary to our expectations, MH1 showed extensive fields of bone resorption over the nasomaxillary complex predominantly in relation to the location of the roots of the anterior teeth (Fig. 2B). This resorption in the MH1 clivus occurred as vertically orientated alternating bands in the depths of the canine fossae between the canines and I^2^, extending upwards toward the inferior border of nasal sill, and between I^1^–I^2^. Areas of the midline clivus in the vicinity of the intermaxillary suture were resorptive, as well as the maxillary furrow (between the nasomaxillary complex and the infraorbital region). Some areas presented resorptive islets with smaller osteoclast lacunae, and so were likely less dynamic. These included: the nasal sill and the infero-lateral border of the nasal aperture. In lateral view, MH1 shows resorption islets on the postero-lateral aspect of the maxillary tuberosity, and small islets over the sphenoid and infratemporal fossa. Bone deposition was identified over: the outer aspects of the orbits, lateral nasal walls, infraorbital region, zygomatico-maxillary region, parts of the mid-clivus including the canine jugum, inter-incisal protuberance as well as portions of the nasal sill and areas lateral to the intermaxillary suture (Fig. 2B). Bone deposition also predominated over most of the antero-lateral aspects of the maxilla, outer aspect of the zygomatic arch and most of the sphenoid and infratemporal fossa. The upper face was markedly depository.

## Discussion

The anatomical relations of the structures forming the nasal sill and palate have been proposed as a model to interpret facial growth in hominins^5^,^6^,^7^,^8^. In this context, we have looked for evidence of these relations in MH1 to provide insights into its effects on facial growth. Like *Au. afarensis* and *Au. africanus*, the nasal cavity entrance of MH1 shows a step-like relationship between the premaxilla and the palate, a characteristic feature of *Australopithecus*^6^ (Fig. 2A). In MH1, the vomer does not contact the premaxilla (Fig. 2A and Fig. S3), a feature also shared with *Au. afarensis* and *Au. africanus* but not with *Paranthropus*. Evidence for the anatomical contact of the vomer in early *Homo* suggests that it was more or less flat^6^,^7^. Functionally, the anterior extension of the vomer onto the premaxilla and the topography of the entrance to the nasal cavity have been associated with the development of a thick palate and other characteristics of the upper face in *Paranthropus*^4^,^5^,^6^. For this genus, the contact of the premaxilla with the vomer facilitated downward drifting of the whole palate, potentially creating a thick palate through differential rates of resorptive remodeling on the nasal floor compared to deposition on the oral surface of the palate^4^,^5^. This anatomical contact and resulting process was associated with a tall posterior face and mandibular ramus in *Paranthropus*, requiring an advanced degree of maxillary rotation^4^,^5^. In contrast, *Au. afarensis* and *Au. africanus* maxillary rotation is reduced, possibly linked to a growth uncoupling of the premaxilla and vomer^4^,^5^. For MH1, the step-like configuration of the palate, premaxilla and vomer contact may have permitted independent growth (drift) of the palate, which, according to this model, would result in its relatively reduced supero-inferior thickness, as shown in Fig. 2A.

A comparison of the developmental characteristics of the face of MH1 with other hominins reveals significant differences (Fig. 2B vs 2C). The facial growth remodeling maps of all sub-adult specimens ascribed to *Au. afarensis* and *Au. africanus* to date, including incomplete or fragmentary maxillae amounting to a total of nine specimens, from two independent studies^4^,^13^, concur in identifying bone deposition as the overwhelming bone surface activity state over the clivus and all other anteriorly-facing aspects of the maxilla^4^,^13^. Such bone deposition is evident throughout different stages of development, ranging from ~3.5 yrs to ~11. 3 yrs^4^,^11^,^12^,^13^, and so likely contributes to the development of the prognathic facial profile characteristic of these taxa^4^,^12^,^13^. The ontogeny of the mesognathic face of MH1 is developmentally distinct, clearly differing from all known *Au. afarensis* and *Au. africanus* specimens in presenting resorptive fields, distributed along vertical tracks over the nasomaxillary complex. Studies of *Paranthropus* (*P. boisei* and *P. robustus*) facial ontogeny also identified bone resorption over the clivus^4^,^12^,^13^ which might be associated with their less projecting maxillae^4^,^11^,^12^ or as a mechanism of local surface sculpting^13^. In *Paranthropus,* resorption is confluent and restricted to the clivus while in MH1 resorptive fields are supero-inferiorly orientated and lie between anterior tooth roots and over the laterally-placed canine fossa resulting in a non-flat anterior maxilla. In this latter feature, MH1 also differs from the *H. erectus/ergaster* specimen KNM-WT 15000 since this specimen presents a flat clivus with no significant corrugations or resorption around tooth roots^21^,^22^. No resorption was identified in the maxilla of KNM-WT 15000^21^ or in preserved maxillae of other early *Homo* specimens such as OH 13 or SK 27^4^,^12^. MH1 however superficially resembles some specimens of *H. sapiens* in the distribution of resorptive fields^18^,^19^. It should be taken into account that the morphogenesis of the *Au. sediba* face at present can only be reconstructed from a single specimen. Despite this, its unique facial anatomy described previously^1^,^2^,^3^ and its facial remodeling pattern reported here have not been identified in any other fossil hominin even in those with similar stages of dental development. Given that the anterior permanent teeth of MH1 were fully erupted and that the canine roots appeared fully closed, it is unlikely that tooth eruption was a key determinant in the unique distribution of remodeling fields on the MH1 lower face.

The *Au. sediba* facial growth remodeling pattern is relevant in the context of determining whether this hypodigm is distinct from *Au. africanus*^1^,^2^,^3^. Although both share a number of morphological characters, they differ in a number of cranial traits including reduced facial prognathism in *Au. sediba*, the conformation of the nasoalveolar region and the infraorbital-zygomatic area^1^,^2^,^3^. Resorptive fields of the MH1 face evidence a dynamic cell-based mechanism that helps to explain aspects of its facial anatomy. The effect of the resorptive fields observed in the clivus of MH1 likely contributed to the development of a less prognathic face in this specimen^1^,^3^ relative to the generally prognathic faces of *Au. africanus*, and to a difference in overall clivus topography with corrugations in the MH1 maxilla. The resorption noted along the lateral border of the maxilla as it contacts the zygomatic to form a furrow in MH1 probably affected the orientation of the infraorbital plate relative to the alveolar plane, which in MH1 forms a right angle whereas in *Au. africanus* is obtuse^1^,^3^. As this resorption in MH1 also limits anteriorward displacement of the zygomatic component of the maxilla, this may explain why the depository face of *Au. africanus* shows flaring zygomatics but not *Au. sediba*. Other morphological features of the face of MH1 such as the curved lateral orbital margin relative to the indented morphology *Au. africanus*^1^,^3^ might be linked to deposition on the lateral aspects of the orbital bones in MH1 whereas the anatomy of *Au. africanus* is associated with resorption in the same areas^11^. Thus, although *Au. sediba* and *Au. africanus* are somewhat close morphologically, our data showing markedly different facial growth remodeling patterns in MH1 compared to all other *Au. africanus* specimens sampled to date, support the notion that these two hypodigms are developmentally distinguishable. These key developmental modifications in MH1 were an important component on the evolutionary novelties present in *Au. sediba*.

A number of features could be associated with the observed changes in facial remodeling of MH1. Brain growth is tightly associated with facial growth through direct structural interactions and molecular signals^23^ that may affect facial prognathism^24^. Facial growth is also influenced by functional matrices, particularly those linked with the masticatory system^9^. Here, *Au. sediba* shows clear differences relative to other australopiths. The shape of the MH1 brain, particularly orbitofrontal shape and organization, is more advanced relative to specimens attributed to *Au. africanus*^25^ and the masticatory system also differs. *Au. sediba* is characterized by temporal lines that are positioned much lower on the skull than in specimens *Au. afarensis* and *Au. africanus* and shows an overall reduction in molar size compared to these taxa^1^. These features are in line with evidence for a different diet in *Au. sediba*^26^ and with general trends for tooth size decrease by 2.0my associated with more warm and humid conditions^27^. The reduced postcanine dentition of MH1 also contributes to its characteristic mesognathy, as it decreases the effects of anteriorward displacements during growth, impacting on how strains from masticatory system loading are distributed over the lower face and so on its distinctive facial remodeling.

**Figure 3:**
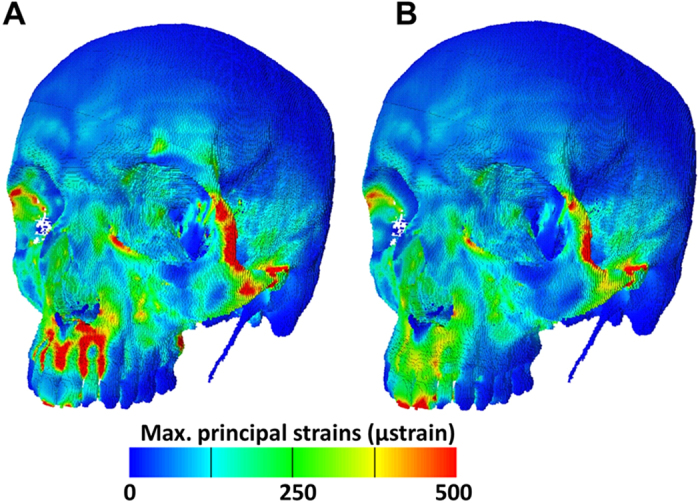
Simulated strain in a human skull. (**a)** Contour map of the maximum principal strains arising from simulated incisor biting in a human. Note the regions of high strain between the incisors and between I^2^ and the canine. **(b)** The high strains noted between the anterior dentition in a) are absent or much reduced when teeth are allocated the same material properties as bone.

Thus, the resorptive bands found over the fossae in the lower face of MH1 are reminiscent of the alternating stripes of high and low strain observed over the roots of these teeth during simulated biting in a human cranial model subjected to finite elements analysis. Figure 3A presents the strains over the surface of a human face arising from simulated biting of 354 N on the central upper incisors (see also Suppl Mat). The alternating vertical stripes of high and low strain over the biting teeth and more distally are potentially associated with the patterning of subsequent remodeling, since bone is well known to adapt to strains^28^,^29^. However, this arrangement of teeth^30^ and bone is a common feature of many mammals and so is insufficient to explain the remodeling pattern difference between MH1 and other hominin taxa mapped to date. But, when teeth and cancellous bone are allocated the same Young’s modulus as cortical bone (17 Gpa) these stripes of alternating strain markedly decrease (Fig. 3B). The implication is that the observed strain distribution arises from an interaction between loading of stiffer teeth held by the periodontal ligament within less stiff overlying bone. This is a hypothesis that merits further testing, through detailed, high resolution, finite element modelling studies of the teeth and surrounding alveolus that simulate the forces applied to the teeth during food acquisition and processing.

How were the teeth of *Au. sediba* loaded?. Little is known of this but it almost exclusively consumed C_3_ foods (dicotyledons and monocotyledons) including tree leaves, fruits, wood and bark, with grasses and sedges, respectively, together with some hard items^26^. The preference of *Au. sediba* for a C_3_ diet despite the availability of C_4_ grasses may be relevant to understanding the unique remodeling of the lower face. Thus, *Procolobus verus* displays a somewhat similar facial remodelling to that of MH1^27^. This forest dwelling monkey consumes primarily young leaves stripped from branches by the jaws but also fruits and seeds and so parallels the diet of *Au. sediba*. The similarity of supero-inferior resorption bands on MH1 and *P. verus* may indicate that biomechanical signals arising from similar loading of the jaws during food acquisition (leaf stripping) underlie the similar distribution of resorptive fields. However, such a conclusion requires further detailed studies of leaf stripping and other primates. The absence of such features in other hominins implies that *Au sediba* may have been derived in this regard.

## Conclusion

The MH1 skull presents a novel combination of primitive and derived features impacting its craniofacial growth. The premaxilla-palatine-vomer contact of MH1 is similar to that of other australopithecines resulting in a supero-inferiorly reduced palatal thickness. On the other hand, the facial bones of MH1 present a distinct pattern of growth remodeling features not yet observed in other *Australopithecus* species. The resorptive fields of the MH1 face may have contributed to its mesognathic face and other facial features perhaps in association with changes in brain shape, diet and/or modes of food acquisition. The result is that MH1 displays a significant modification in facial ontogeny at 1.9 my being developmentally distinguishable from *Au. africanus*. Given the number of craniodental features that link *Au. sediba* with early *Homo*^1^,^2^,^3^, and given that the facial remodeling pattern of MH1 has not been recorded in early *Homo*, these data suggest that developmental changes in the face occurred independently of other craniodental features (e.g. reduction in tooth size) supporting a possible specialization in the adaptive niche of *Au. sediba* as indicated by its diet^26^.

## Methods

### Preparation of MH1

The MH1 skull was cleaned using 70% ethanol to remove debris prior to moulding. Curatorial procedures for this specimen by the preparators at the University of the Witwatersrand did not include the use of any adherents, facilitating the casting of MH1 skull. All cleaned surfaces were replicated using only the silicone based Coltene President light body consisting of a base mixed in equal proportion with the catalyst (green) (Fig. S2). Two replicas of each area of the face and upper skull were made. Each replica was examined under a light microscope to assess the quality of the replica. After replicas were made, the entire MH1 skull and breccias were examined using a magnifying glass to remove any casting material that may have lodged into any of these areas. Any material identified was removed using a wooden toothpick. Positives were made using Devcon 5-minute Epoxy (ITW Devcon, Danvers, MA). Uncoated positives were examined by an EVO 50 scanning electron microscope (Carl Zeiss, Thornwood, NY) in variable pressure secondary electron emission mode (15 kV, 200 pA current, 10 mm working distance, 100 Pa pressure).

### Identification of depository vs resorptive bone surfaces and synchrotron scanning

Depository surfaces are identified by features associated with the activities of osteoblasts. Such surfaces are relatively isotropic containing bundles of mineralized collagen fibers and remnants of osteocyte lacunae –bone cell spaces- and capillaries^12^ (Fig. 1 and Fig. S1). Resorption areas in contrast show irregular surfaces containing evidence of the activity of bone removing cells, the osteoclasts, that form anisotropic surfaces characterized by resorption bays called Howship’s lacunae^10^ (Fig. 1 and Fig. S1). Synchrotron scanning of MH1 was reported in ref. 25.

### Finite element model

A CT scan of an adult male was used in this study. The head was scanned at York Teaching Hospital (York, UK) using a Siemens 16-channel multidetector CT scanner equipped with a STRATON tube (Siemens Somatom Sensation 16, Siemens Healthcare, Erlangen, Germany). Voxel size was 0.48 × 0.48 × 0.7 mm. The image stacks were exported as DICOM files. The cranium was reconstructed using the visualisation software tool, Avizo v. 7.0.1 (Visualization Sciences Group, Burlington, USA). A combination of automatic thresholding and manual detailing was used to segment cortical and trabecular bone, and teeth. The final volume data were resampled to isometric voxels with side length 0.48 mm, exported as BMP stacks and converted into a FE mesh of 3,505,131 eight-noded elements, by direct voxel conversion. Values of Young’s modulus were allocated as follows; 17GPa to cortical bone, 56 MPa to cancellous bone and 50 GPa to teeth (Fig. 3A) or 17 GPA to teeth (Fig. 3B), and a Poisson’s ratio of 0.3 was used for all materials. Applied muscle forces (left temporalis = 156.3 N; right temporalis = 169.4 N; left masseter = 234.7 N; right masseter = 223.9 N; left medial pterygoid = 137.1 N; right medial pterygoid = 126.8N) were estimated from the observed CSAs of the jaw-elevator muscle^31^. Force vectors were oriented towards the centroids of the mandibular muscle insertions observed in the CT. Kinematic constraints were applied to the borders of the central upper incisors in the vertical axis, and to the antero-superior surfaces of both mandibular fossae in the vertical, horizontal and antero-posterior axes. Model pre- and post-processing was performed using our custom-made software VOX-FE^32^. Contour maps of the resulting maximum principal strains are presented in Fig. 3A,B. A horizontal line drawn on the left side, a little above the alveolar margins of the anterior teeth was used to sample principal strain magnitudes.

## Additional Information

**How to cite this article**: Lacruz, R. S. *et al.* Distinct growth of the nasomaxillary complex in *Au. sediba*. *Sci. Rep.* **5**, 15175; doi: 10.1038/srep15175 (2015).

## Supplementary Material

Supplementary Information
