# Supplementary material for: Distinct growth of the nasomaxillary complex in Au. sediba
Source: Sci Rep. 2015 Oct 15;5:15175. doi: 10.1038/srep15175 (PMC4606807; doi:10.1038/srep15175)
Supplement: Supplementary Information [file srep15175-s1.pdf]

Supplementary Information:

Distinct growth of the nasomaxillary complex in *Au. sediba*

Rodrigo S. Lacruz, Timothy G. Bromage, Paul O'Higgins, Viviana Toro-Ibacache, Johanna Warshaw, Lee R. Berger

Supplementary Methods: Figure S1: Identification of bone deposition vs bone resorption: Bone growth remodeling consists of coordinated bone deposition and resorption to effect increasing size and changing shape during development[1-5]. Thus, knowledge of the mode and disposition of remodeling activities can be informative with respect to the bone cortical drifts displacements and of skeletal elements[1-5]. Remodeling activities maintained over long growth periods contribute to determining overall growth direction of the face whereas shorter periods of remodeling activity likely result in localized changes in morphology[6-8].

Growth remodeling activity was identified in the microanatomy of the outer surface of the facial bones of MH1 using high-resolution replicas examined and mapped by scanning electron microscopy (SEM). Resorbing areas show irregular, or vermiculate-like resorption spaces surfaces that demonstrate evidence of the activity of bone removing cells, the osteoclasts. Osteoclasts form these anisotropic resorption bays called Howship's lacunae[9]. There are variations of this surface, sometimes appearing as well rounded resorption cavities (Fig. 1) and other times having the more vermiculate appearance (Suppl Fig S1 panels a,b). Depository surfaces on the other hand are identified by features associated with the activities of osteoblasts. Such surfaces are relatively isotropic containing arrays, or bundles of mineralized collagen fibers and remnants of osteocyte lacunae –bone cell spaces- and capillaries laid down to support osteoblast activity, the latter forming what has been termed "intervascular ridging" bone[9] (Fig S1, panels c,d).

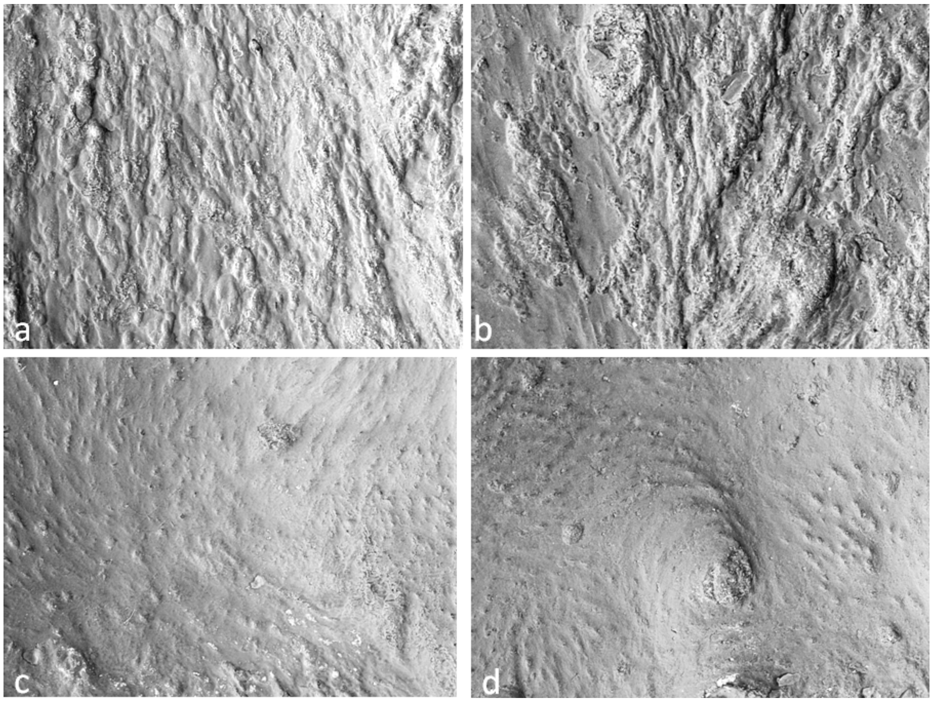

Figure S1: **Bone surface remodelling of the MH1 face**. **a)** Area of the left side of maxilla of the I^1-I^2 fossa (FW 1100 µm). The bulge of the I^1 root was depository whereas resorption can be seen in the fossa. **b)** Area of resorption near the mid-clivus in close proximity to intermaxillary suture (651µm). **c)** Deposition identified over the left canine jugum (FW 890µm).
d) Deposition identified on the inferior temporal fossa (FW 1000 µm).

Figure S2: Preparation and imaging of MH1: The MH1 skull was cleaned using 70% ethanol to remove debris prior to moulding. Curatorial procedures for this specimen by the preparators at the University of the Witwatersrand did not include the use of any adherents, facilitating the casting of MH1 skull. All cleaned surfaces were replicated using only the silicone based Coltene President light body consisting of a base mixed in equal proportion with the catalyst (green) (Fig S2). Two replicas of each area of the face and upper skull were made. Each replica was examined under a light microscope to assess the quality of the replica. After replicas were made, the entire MH1 skull and breccias were examined using a magnifying glass to remove any casting material that may have lodged into any of these areas. Any material identified was removed using a wooden toothpick. Positives were made using Devcon 5-minute Epoxy (ITW Devcon, Danvers, MA). Uncoated positives were examined by an EVO 50 scanning electron microscope (Carl Zeiss, Thornwood, NY) in variable pressure secondary electron emission mode (15kV, 200pA current, 10mm working distance, 100Pa pressure).

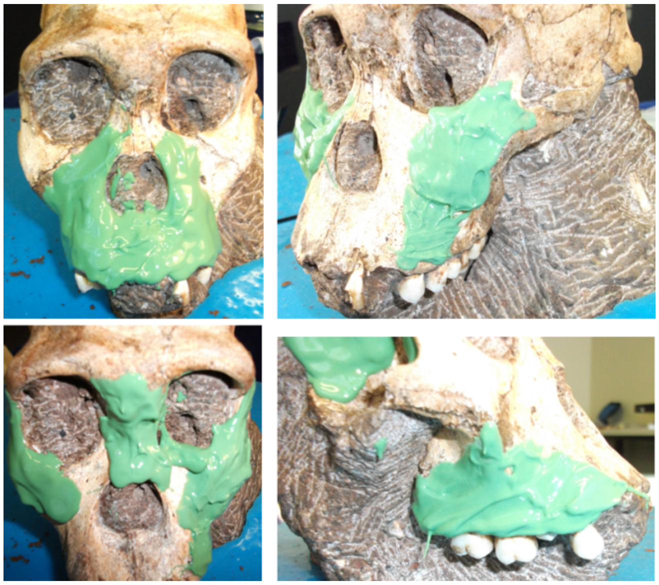

Figure S2

Figure S3: Synchrotron generated image of vomer of MH1: The MH1 skulls had been previously imaged using high energy synchrotron radiation as described in reference 10. Shown here is a virtual section (top view) on the horizontal plane of the *Au. sediba* (MH1) skull. The images show an area near the anterior-most projection of the inferior surface of the vomer. The palatal suture can also be appreciated in this image. The vomer (V) does not contact the premaxilla as evidenced by the gap identified between anterior portion of the vomer (AV) and nasal aperture (NA). The vomer and nasal aperture were highlighted (right image) for convenience.

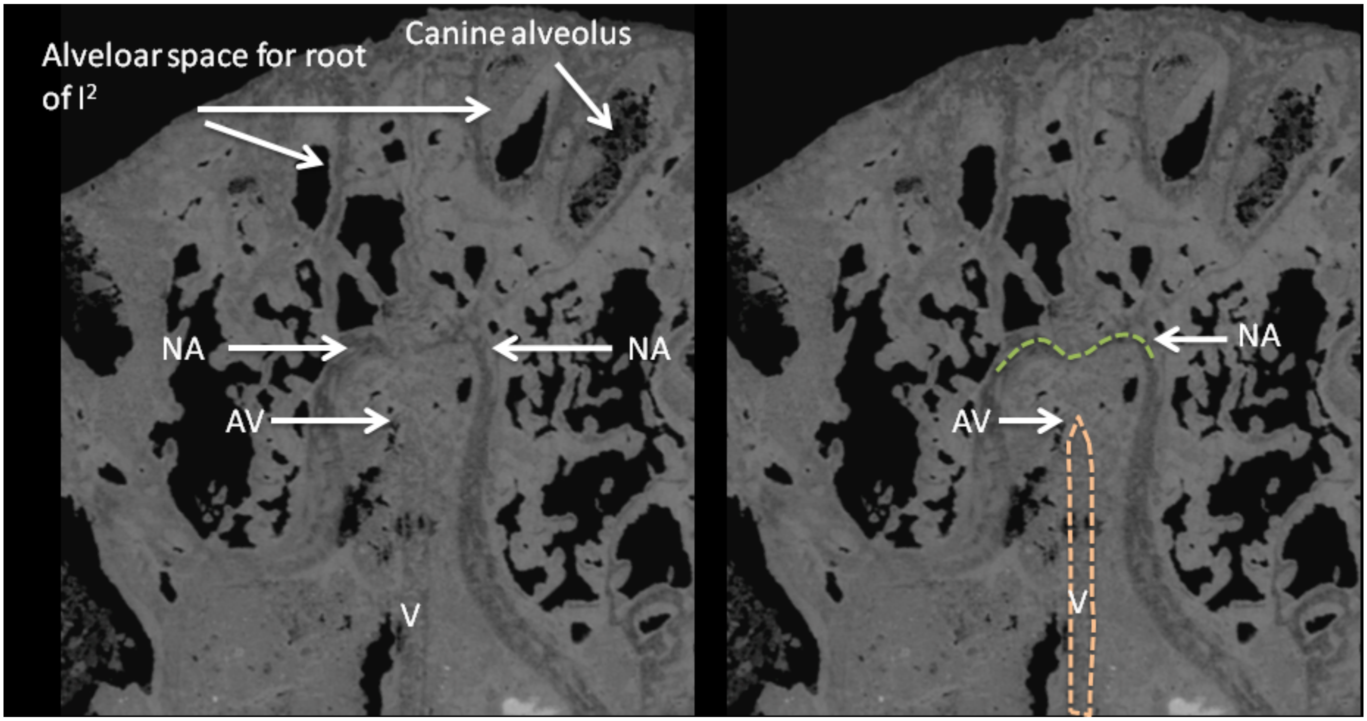

Figure S3.

Figure S4: Synchrotron generated image of the palate of MH1: The MH1 skulls had been previously analyzed using high energy synchrotron radiation as described in reference 10. **Figure S4** shows a virtual sagittal section of the MH1 skull. This virtual section of the MH1 skull was taken at a point near the mid-line. Both synchrotron and CT-data show a step-like entrance into the nasal floor. This configuration is also evident lateral to the incisive foramen following the scheme for interpreting the intranasal anatomy described in reference 11. Image was colour coded for convenience.

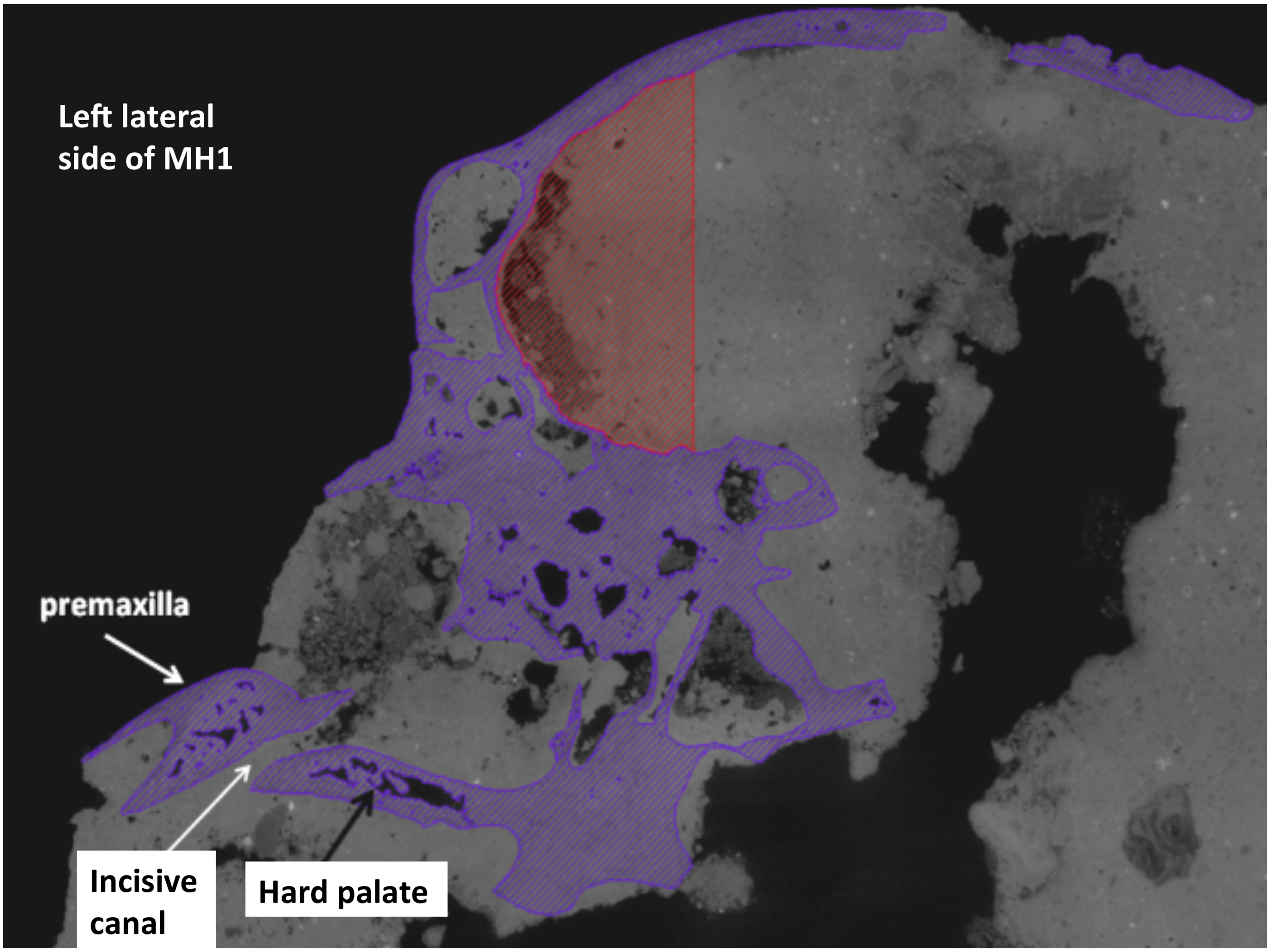

Figure S4.

Figure S5: Finite element model of a human cranium: strains between anterior dentition

A CT scan of an adult male was used in this study. The head was scanned at York Teaching Hospital (York, UK) using a Siemens 16-channel multidetector CT scanner equipped with a STRATON tube (Siemens Somatom Sensation 16, Siemens Healthcare, Erlangen, Germany). Voxel size was 0.48 x 0.48 x 0.7 mm. The image stacks were exported as DICOM files. The cranium was reconstructed using the visualisation software tool, Avizo v. 7.0.1 (Visualization Sciences Group, Burlington, USA). A combination of automatic thresholding and manual detailing was used to segment cortical and trabecular bone, and teeth. The final volume data were resampled to isometric voxels with side length 0.48 mm, exported as BMP stacks and converted into a FE mesh of 3,505,131 eight-noded elements, by direct voxel conversion. Values of Young's modulus were allocated as follows; 17GPa to cortical bone, 56 MPa to cancellous bone and 50 GPa to teeth (Fig 3a) or 17GPA to teeth (Fig3b), and a Poisson's ratio of 0.3 was used for all materials. Applied muscle forces (left temporalis=156.3 N; right temporalis=169.4 N; left masseter=234.7 N; right masseter=223.9 N; left medial pterygoid=137.1 N; right medial pterygoid=126.8N) were estimated from the observed CSAs of the jaw-elevator muscles[12]. Force vectors were oriented towards the centroids of the mandibular muscle insertions observed in the CT. Kinematic constraints were applied to the borders of the central upper incisors in the vertical axis, and to the anterosuperior surfaces of both mandibular fossae in the vertical, horizontal and antero-posterior axes. Model pre- and post-processing was performed using our custom-made software VOX-FE[13]. Contour maps of the resulting maximum principal strains are presented in Figure 3a,b. A horizontal line drawn on the left side, a little above the alveolar margins of the anterior teeth was used to sample principal strain magnitudes. In Figure S5, below, these magnitudes are plotted against distance in voxels from the midline (X). Note the resulting curves are somewhat jagged as is expected when using voxel based models. The difference between the maximum and minimum strain at each point was computed and plotted as a dashed line in the same Figure. LI1 and LI2, indicate the positions of the mid points of the upper left first and second incisors respectively. On average, maximum and minimum principal strains are higher, between the teeth, as is also indicated by the contour map of Figure 3a. The differences between the principal strains indicate that tension predominates between incisors (absolute value of maximum principal strain > minimum). Net tension has previously been associated with the absence of bone and bone resorption (e.g. in the incus; ref. 14) and may therefore explain the presence of the resorptive fields observed in MH1 and *P. verus* (Fig S6) as resulting from the mechanical interaction between tooth root and overlying alveolar bone during food acquisition and processing by the jaws and teeth.

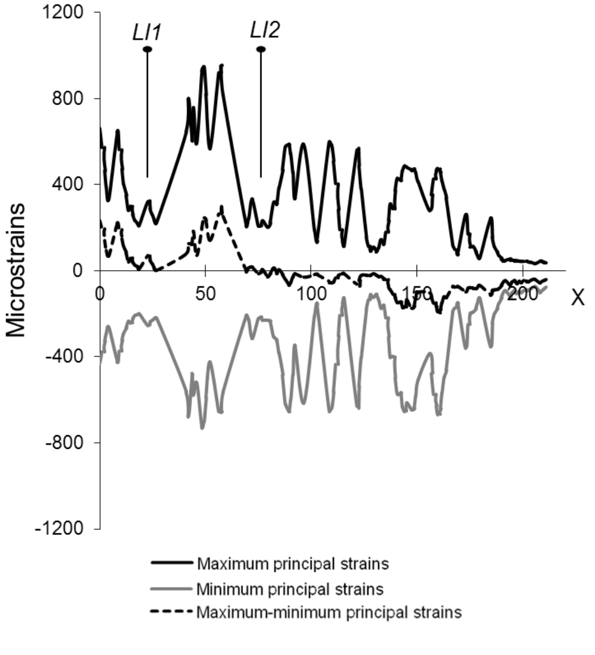

Figure S5. Plot of principal strain magnitudes against distance in voxels from the midline as described in the text.

Suppl Material References

1. Enlow DH, Hans MG (1996) *Essentials of facial growth* (W.B. Saunders, Philadelphia).
2. Enlow DH (1966) A morphogenetic analysis of facial growth. *Am J Orthod* 52: 283-299
3. Sarnat BG (1997) Postnatal facial growth: Some methods and assessments. *Fundamentals of craniofacial growth*, eds Dixon AD, Hoyte D, Ronning O (CRC Press, Boca Raton, Florida), pp 137-154.
4. Hoyte DAN (1997) Bone remodeling in the craniofacial region. *Fundamentals of craniofacial growth,* eds Dixon AD, Hoyte D, Ronning O (CRC Press, Boca Raton, Florida), pp 427-442.
5. Kurihara S, Enlow DH, Rangel RD (1980) Remodeling reversals in the anterior parts of the human mandible and maxilla. *Angle Orthod* 50, 98-106.
6. Bromage TG (1989) Ontogeny of the early hominid face. *J Hum Evol* 18:751-773.
7. McCollum MA (2008) Nasomaxillary remodeling and facial form in robust *Australopithecus*: a reassessment. *J Hum Evol* 54: 2-14.
8. Lacruz RS *et al*. (2013) Facial morphogenesis of the earliest Europeans, *PLoS ONE*, 8(6): e65199.
9. Bromage TG, Boyde A (2008) Bone growth remodeling of the early human face. *Essentials of facial growth.* eds. Enlow DH, Hans MG (WB Saunders, Philadelphia), pp 319-342.
10. Carlson KJ *et al*. (2011) The endocast of MH1, *Australopithecus sediba*, *Science* 333, 1402-1407.
11. McCollum MA (2000) Subnasal morphological variation in fossil hominins: a reassessment based on new observations and recent developmental findings. *Am J Phys Anthropol* 112: 275-283.
12. Weijs W, Hillen B (1984) Relationship between the physiological cross-section of the human jaw muscles and their cross-sectional are in computer tomograms. *Acta Anat* 118, 129-138.
13. Liu. J., *et al.* (2011) The application of muscle wrapping to voxel-based finite element models of skeletal structures. *Biomech Model Mechanobiol* 11**,** 35- 47.
14. Oxnard CE *et al*., (1995) *Bone Structure and Remodelling*, eds Odegaard A, Weinans H (World Scientific, Singapore, 1995) pp. 105-125
